# Internet Addiction in Constipated Adolescents

**DOI:** 10.5152/tjg.2023.22190

**Published:** 2023-03-01

**Authors:** Sevim Çakar, Gülin Eren

**Affiliations:** 1 The Division of Pediatric Gastroenterology and Nutrition, Department of Pediatrics, University of Health Sciences Dr. Behçet Uz Children’s Hospital, İzmir, Turkey

**Keywords:** Constipation, internet addiction, adolescent

## Abstract

**Background:**

This study aims to assess internet addiction among adolescents with functional constipation and its relationship with sociodemographic and family factors.

**Methods:**

In this case–control study, 57 and 35 adolescents with and without functional constipation, respectively, were recruited. Functional constipation was diagnosed by applying the Rome IV criteria. Previously validated Young’s Internet Addiction Test-Short Form and Parent–Child Internet Addiction Test were completed, and sociodemographic data forms were filled out by all participants.

**Results:**

According to the Parent–Child Internet Addiction Test questionnaire, 8.8% (n = 5) of the adolescents with FC suffered from internet addiction, whereas none of the healthy adolescents had internet addiction. The Young’s Internet Addiction Test-Short Form survey reported internet addiction among 19.3% (n = 11) of the constipated adolescents and 17.1% (n = 6) of the healthy peers. The ratio of adolescents with limited symptoms and internet addiction in the functional constipation group was as high as 40.4%, according to Young’s Internet Addiction Test-Short Form, and adolescents with functional constipation accompanied by pathological and problematic internet use was 24.6%, according to Parent–Child Internet Addiction Test. Although there was no significant difference in the internet addiction ratio between functional constipation and controls, our findings indicated that adolescents with functional constipation were suffering from high internet addiction rates.

**Conclusion:**

The evaluation of Young’s Internet Addiction Test-Short Form and Parent–Child Internet Addiction Test surveys showed high internet usage problems, 40.4% and 24.6% in adolescents with functional constipation. According to adolescents’ self-assessment, the internet addiction rate is higher but not significantly higher than their healthy peers. Therefore, internet addiction should be considered while treating adolescents with functional constipation, and clinicians should consider the opinions of adolescents besides their parents.

## Introduction

Functional constipation (FC) is a global problem associated with lifestyle factors in children and adolescents, with a prevalence of 10% approximately.^1^ Adolescents with FC are suffering from significant behavioral and emotional problems, and the etiology of FC is mostly unknown.^2^

Internet behavior disorder is most relevant to adolescents as they more frequently access the internet than other age groups.^3,4^ Adolescents use the internet as a source of information about interests, daily events, school assignments, hobbies, health, and sexuality concerns. Communicating with peers is possible through online activities. Learning faster, developing skills in critical thinking and decision-making, exercising one’s self-control, considering different opinions, expressing one’s attitudes and tolerance are positive influences of internet use. On the other hand, relevant academic papers about the excessive use of the internet have been increasing. With rapidly developing technologies, the COVID-19 pandemic, and the new daily life, internet usage has moved into homes, schools, and businesses. As a result, the prevalence of internet addiction (IA) has been increasing sharply. It is an internet behavior disorder characterized by poorly controlled internet use that can lead to impulse control disorders.

Prevalence statistics of IA in adolescent age widely ranged from 2% to 20% and higher in Asia.^5-7^ Research has confirmed the link between IA and depression, anxiety, sleep problems, obesity, increased use of alcohol and tobacco, and many problems with physical health.^8-13^

Some studies reported that heavy internet use changes lifestyle-related factors and causes an adverse impact on growth and development.^13,14^ Moreover, loss of control and excessive internet use lead to a sedentary position, decreased physical inactivity, delayed defecation, and increased food intake with poorer diet quality (eat smaller meals, have less of an appetite, skip meals, and snack more).^14^ Considering that the dietary habits are formed during childhood, and they tend to be carried on throughout adulthood also, FC is shown to be associated with obesity, a low dietary fiber intake, and behavioral–emotional problems in adolescents; IA can contribute to FC among children and adolescents.^15^

Thus, we evaluated IA in adolescents with FC and hypothesized that the ratio of IA is higher among adolescents with FC than among healthy controls.

## Materials and Methods

### Participants

This case–control study was conducted in a tertiary public hospital between January 1, 2021, and July 1, 2021. Adolescents with FC aged between 13 and 18 years and their parents who agreed to participate in the study were recruited from the outpatient clinic of the pediatric gastroenterology department. A pediatric gastroenterologist diagnosed FC by applying the Rome IV criteria. The control group consisted of healthy adolescents without FC and their parents. Patients with a known genetic disease, obesity, hypertension, history of head trauma, and endocrine, neurologic, gastroenterological, or psychiatric disorders were excluded.

### Procedure

The participants were informed about the study. Then, the written consent of the parents and patients who agreed to participate in the study was obtained. Finally, the researchers collected data through face-to-face interviews with the parent and adolescents at the outpatient clinic. The participants took an average of 15-20 minutes to complete the questionnaire form.

### Measures

The World Health Organization, the Diagnostic and Statistical Manual of Mental Disorders (DSM-5), and the International Classification of Diseases (ICD-11) have not recognized excessive internet use as a disorder. Clinical diagnostic criteria have not been established to classify IA. Thus, we administered a previously validated Young’s Internet Addiction Test-Short Form (YIAT-SF) to adolescents and Parent–Child Internet Addiction Test (PCIAT) to parents along with sociodemographic forms to all participants.^16-19^

### Parent–Child Internet Addiction Scale

The PCIAT was developed by Young to determine families’ views about the internet use of their children.^19^ Eşgi^18^ adapted the PCIAT to Turkish patients using a Likert scale consisting of 20 items and 4 subscales.^18^ If the PCIAT score was higher than 80, between 79 and 50, and lower than 49, then the patients were categorized as internet-addicted, children with limited symptoms, and children with average internet use, respectively.

### Young’s Internet Addiction Test-Short Form

One of the most frequently used questionnaires is Young’s Internet Addiction Test (IAT), though still discussed controversially. Pawlikowski revealed a short version of the IAT, which consists of 12 items and retained the original 5-point Likert scale.^17^ Kutlu et al^16^ adapted such a version to Turkish patients. The overall score of the survey is recommended when examining an individual’s tendency to or degree of IA. The cut-off score of the YIAT-SF for problematic and pathological internet use was >30 and >37, respectively.

### Supplementary Form

The researchers prepared this form, which was filled out during the interview. The form included information about sociodemographic characteristics, duration of constipation, medication for constipation and its response, presence of delaying defecation, presence of fecal and urinary incontinence, amount of fruit and vegetable consumption, and regular exercise (at least 5 days a week, 20 minutes a day) habits.

### Ethical Consideration

Ethical approval was obtained from the University of Health Sciences, Dr. Behçet Uz Children’s Hospital Ethics Committee. The respondents participated in the study voluntarily. The confidentiality and anonymity of the subject were maintained throughout the study.

### Analysis

The sample size was calculated as 34 with a 5% level of significance and 80% power based on the assumption that 10% of children with constipation would have IA. Statistical Package for the Social Sciences (SPSS) software version 21.0 (IBM Corp.; Armonk, NY, USA) was used for the analysis. The Kolmogorov–Smirnov or Shapiro–Wilk test was used to determine the distribution of variables. The independent sample Student’s *t*-test and Mann–Whitney *U* test were used to compare the normally and non-normally distributed data sequentially. Chi-square tests were used to compare the differences in categorical variables between groups. When the significance was observed, pairwise comparisons were performed using chi-square tests with Bonferroni correction. The possible factors identified with univariate analyses were further analyzed for the binary logistic regression analysis to determine independent factors. The Hosmer–Lemeshow goodness of fit statistics assessed model fit. Statistical significance was regularly assigned to *P*-values less than .05.

## Results

The median age of the FC and control groups was 14 years (IQR = 3 years). The median duration of constipation was 12 months (IQR = 30.5 months). In the FC group, 21.1% (n = 12) of the patients used pharmacological treatment for constipation. However, the treatment was partially effective on only 14.0% (n = 8). Moreover, 7% (n = 4) and 3.5% (n = 2) of the patients in the FC group had fecal and urinary incontinence, respectively. In the control group, none of the adolescents had fecal incontinence, whereas 5.7% (n = 2) had urinary incontinence. The FC and control groups had no difference in terms of the mother’s education and employment status (*P* = .900 and *P* = .169). The ratio of delaying defecation was not significantly different between the two groups (*P* = .216), with 56.1% (n = 32) and 42.9% (n = 15) for the FC group and control group, respectively. The daily vegetable consumption was statistically lower in the FC group (*P* = .039). Table 1 presents the sociodemographic features.

Table 2 provides the mean scores of the YIAT-SF and PCIAT in the FC and control groups.

The YIAT-SF survey showed that 19.3% (n = 11) of the FC group and 17.1% (n = 6) of the healthy peers suffered from IA. This finding was not statistically higher in the FC group (*P* = .649). According to the PCIAT, 8.8% (n = 5) of the FC group was determined to have IA, whereas none of the healthy adolescent control group had IA. Table 3 presents the results of PCIAT and YIAT-SF in FC and control groups. The ratio of the 3 groups according to PCIAT was significantly different between the FC group and controls (*P *= .037). But post hoc analysis revealed that the high ratio of children with limited symptoms in the control group is the source of the difference (15.8% vs. 34.3%). In pairwise comparisons, no statistical significance was found in the ratio of IA and average users with controls (*P *= .167).

The logistic regression model was formed to assess the effect of daily vegetable consumption and IA according to PCIAT score on the presence of FC, and the vegetable consumption level was the only independent factor for FC in adolescents (odds ratio [OR] = 0.501, 95% CI = 0.284-0.884).

## Discussion

To our knowledge, no research has demonstrated IA among patients with FC and the association between IA and FC in adolescents. Our study is the first in the literature to investigate this topic. Although the parents’ survey questionnaire was not statistically significant, we indicated a higher rate of IA in the FC group with 8.8%, compared with no internet-addicted adolescents in the control group. The self-assessment test survey also indicated a high IA rate in the FC group with 19.3%, though it was similar to the control group. When we asked the adolescents themselves, not the parents, the rate of IA was more than twice. That can be explained by the fact that they isolate themselves from the outside world in adolescence and stay relatively free of parental supervision. As adolescents know themselves better, they can provide us with more accurate information about addiction.

The definition of IA has been extensively debated and developed over the past 20 years, with prevalence rates at 1%-5% reported in adolescent-age studies.^4,20-22^ Dong et al^23,^ reported 2.68% IA, with 33.37% problematic internet use during the COVİD-19 pandemic, whereas Adiele and Olatokun^24^ found an IA rate of 3.3% among adolescents. According to Young’s IAT (1999), 1.5% of Greek and 1.6% of Finnish adolescents suffered from IA.^25,26^ The rates of IA are often high in Asian countries with 10.9%.^27^ Similarly, the IA rate of our study population stands out as extremely high.

Young claims that IA is a broad term that covers various behaviors and impulse control problems.^28^ Internet use is also associated with other psychiatric disorders, such as depression and low self-esteem.^6,29,30^ Moreover, IA has been described as uncontrollable and harmful internet use and was found to be a predictor of the development of sleep problems, eating disorders, and obesity.^8,9,12^ Eating disorders can lead to a liquid- and fiber-poor diet, resulting in constipation. Functional constipation is a global problem and depends on many lifestyle factors, with a prevalence of approximately 9%-12.9% in childhood.^1,31^ Considering the adolescent period, a higher prevalence of FC with 24.9% was reported in a Brazilian study.^15^ Furthermore, adolescents with FC are suffering from significant behavioral and emotional problems, including IA withdrawal, somatic complaints, anxiety, and depression, the same problems that accompany IA.^2,6,30^ Therefore, FC and IA coexistence can be expected, and IA may be one of the underlying factors in FC. Our results support this hypothesis as the ratio of limited symptoms and IA according to YIAT-SF was as high as 40.4% in adolescents with FC while 24.6% the of FC group with pathological and problematic internet use according to PCIAT.

Loss of control over food intake during internet use and increased consumption of low-fiber junk food diet in front of the screen may be contributing factors to FC in youths with IA. Consistent with this, vegetable consumption was low in our population with FC.

Regarding physical activity, no randomized studies have evaluated its effect on childhood constipation.^15,32^ Also, our data did not show a difference in performing routine daily exercise between adolescents with FC and the healthy group. On the other hand, excessive internet use causing a sedentary position decreased physical activity, and delayed defecation expected in adolescents with IA may contribute to FC.

As a result, the European Society for Pediatric Gastroenterology (ESPGHAN) and the North American Society for Pediatric Gastroenterology (NASPGHAN) recommended that mental health providers may help children with constipation and behavioral abnormalities though they do not have them.^31^ Increasing physical activity and healthy eating routines after the diagnosis and treatment of IA may provide a successful interventional approach for certain individuals with constipation.

## Conclusions

We indicated that adolescents with FC are suffering from IA at higher rates than the healthy population. Also, low vegetable consumption is an important independent risk factor in FC.

We propose that psychosocial assessment in adolescents with FC is essential. Moreover, parents, healthcare professionals, and education professionals should accept IA as a public health problem. Thus, IA should be considered when treating adolescents with FC, and further studies with larger numbers of patients will help clarify the unknown.

## Figures and Tables

**Table 1.** Sociodemographic Features of the Study Population

	FC Group, n (%)	Control Group, n (%)	*P*
Gender			
Male	20 (35.1)	15 (42.9)	*.456*
Female	37 (64.9)	20 (57.1)	
Having own smartphone	51 (89.5)	34 (97.1)	*.178*
Having own computer	32 (56.1)	21 (68.6)	*.236*
Routine daily exercise	7 (12.3)	5 (14.3)	*.782*
Daily vegetable consumption (portion/day)	0: 27 (47.4) 1: 15 (26.3) 2: 15 (26.3) 3: -	0: 9 (25.7) 1: 12 (34.3) 2: 13 (37.1) 3: 1 (2.9)	***.039***
Daily fruit consumption (portion/day)	0: 20 (35.1) 1: 24 (42.1) 2: 10 (17.5) 3: 3 (5.3)	0: 8 (22.9) 1: 20 (57.2) 2: 6 (17.1) 3: 1 (2.9)	*.497*

FC, functional constipation.

Routine daily exercise, at least 5 days a week, 20 minutes a day. Bold-italic *P*-value has statistical significance.

**Table 2.** The Mean Scores of the Questionnaires

Questionnaire Score	FC Group (Mean)	Control Group (Mean)	*P*
PCIAT score	35.6 ± 22.3	37.2 ± 22.8	*.729*
YIAT-SF score	28.0 ± 8.5	26.6 ± 8.6	*.452*

FC, functional constipation; PCIAT, Parent–Child Internet Addiction Test; YIAT-SF, Young’s Internet Addiction Test-Short Form.

**Table 3.** Internet Addiction Rates According to Surveys

Questionnaire Group	FC Group, n (%)	Control Group, n (%)	*P*
PCIAT internet addicted (score ≥80) PCIAT with limited symptoms (score 79-50) PCIAT average user (score ≤49)	5 (8.8) 9 (15.8) 44 (75.4)	0 12 (34.3) 23 (65.7)	***.037***
YIAT-SF pathological internet user (score >37) YIAT-SF problematic internet user (score 31-37) YIAT-SF average user (score ≤30)	11 (19.3) 12 (21.1) 34 (59.6)	6 (17.1) 5 (14.3) 24 (68.6)	*.649*

FC, functional constipation; PCIAT, Parent–Child Internet Addiction Test; YIAT-SF, Young’s Internet Addiction Test-Short Form. Bold-italic *P*-value has statistical significance.
